# High-Throughput Sequencing of Gastric Cancer Patients: Unravelling Genetic Predispositions Towards an Early-Onset Subtype

**DOI:** 10.3390/cancers12071981

**Published:** 2020-07-21

**Authors:** Julita Machlowska, Przemysław Kapusta, Jacek Baj, Folkert H. M. Morsink, Paweł Wołkow, Ryszard Maciejewski, G. Johan A. Offerhaus, Robert Sitarz

**Affiliations:** 1 Center for Medical Genomics-OMICs high-throughput technologies (OMICRON) project, Jagiellonian University Medical College, 31-034 Kraków, Poland; julita.machlowska@gmail.com (J.M.); przemyslaw.kapusta@uj.edu.pl (P.K.); pawel.wolkow@uj.edu.pl (P.W.); 2 Department of Human Anatomy, Medical University of Lublin, 20-090 Lublin, Poland; jacek.baj@umlub.pl (J.B.); maciejewski.r@gmail.com (R.M.); 3 Department of Pathology, University Medical Center Utrecht, 3584 CX Utrecht, The Netherlands; f.h.m.morsink@umcutrecht.nl (F.H.M.M.); g.j.a.offerhaus@umcutrecht.nl (G.J.A.O.); 4 Department of Surgery, Center of Oncology of the Lublin Region St. Jana z Dukli, 20-090 Lublin, Poland

**Keywords:** gastric cancer, conventional gastric carcinoma, early-onset subtype, next-generation sequencing, pathogenicity, early diagnosis, molecular biomarkers

## Abstract

Background: Gastric cancer is the fourth most common cause of cancer-related death. Currently, it is broadly accepted that the molecular complexity and heterogeneity of gastric cancer, both inter- and intra-tumor, display important barriers for finding specific biomarkers for the early detection and diagnosis of this malignancy. Early-onset gastric cancer is not as prevalent as conventional gastric carcinoma, but it is a preferable model for studying the genetic background, as young patients are less exposed to environmental factors, which influence cancer development. Aim: The main objective of this study was to reveal age-dependent genotypic characteristics of gastric cancer subtypes, as well as conduct mutation profiling for the most frequent alterations in gastric cancer development, using targeted next-generation sequencing technology. Patients and methods: The study group included 53 patients, consisting of 18 patients with conventional gastric cancer and 35 with an early-onset subtype. The DNA of all index cases was used for next-generation sequencing, employing a panel of 94 genes and 284 single nucleotide polymorphisms (SNPs) (TruSight Cancer Panel, Illumina), which is characteristic for common and rare types of cancer. Results: From among the 53 samples processed for sequencing, we were able to identify seven candidate genes (*STK11*, *RET, FANCM, SLX4, WRN, MEN1,* and *KIT*) and nine variants among them: one splice_acceptor, four synonymous, and four missense variants. These were selected for the age-dependent differentiation of gastric cancer subtypes. We found four variants with C-Score ≥ 10, as 10% of the most deleterious substitutions: rs1800862 (*RET*), rs10138997 (*FANCM*), rs2230009 (*WRN*), and rs2959656 (*MEN1*). We identified 36 different variants, among 24 different genes, which were the most frequent genetic alterations among study subjects. We found 16 different variants among the genes that were present in 100% of the total cohort: *SDHB* (rs2746462), *ALK* (rs1670283), *XPC* (rs2958057), *RECQL4* (rs4925828; rs11342077, rs398010167; rs2721190), *DDB2* (rs326212), *MEN1* (rs540012), *AIP* (rs4930199), *ATM* (rs659243), *HNF1A* (rs1169305), *BRCA2* (rs206075; rs169547), *ERCC5* (rs9514066; rs9514067), and *FANCI* (rs7183618). Conclusions: The technology of next-generation sequencing is a useful tool for studying the development and progression of gastric carcinoma in a high-throughput way. Our study revealed that early-onset gastric cancer has a different mutation frequency profile in certain genes compared to conventional subtype.

## 1. Introduction

Gastric cancer (GC) is a heterogeneous disease with a wide range of molecular and genetic alterations that contribute to the development and progression of this malignancy [1,2]. Statistical investigations have revealed that GC is the fourth most common cause of cancer-related death worldwide, with a median overall survival of ≤12 months for an advanced stage [3]. Early-onset gastric cancer (EOGC) (onset in individuals ≤45 years old) is not as common as conventional gastric carcinoma (CGC); besides, fewer than 10% of patients are affected by GC before 45 years of age [4]. Recent investigations on EOGC have attempted to clarify the genetic background of GC, as young patients are less susceptible to environmental agents that predispose individuals to cancer [5].

GC expands through a round of well-established and distinguishable steps: inflammation, atrophy, intestinal metaplasia, dysplasia, and carcinogenesis. GC pathogenesis is closely related to the diet, environment, *H. pylori* infection, and genetic alterations [6,7,8,9]. The recognizable patterns of GC incorporate genetic alterations among various factors: cell cycle regulators, agents that regulate apoptosis, microsatellite instability, multidrug resistance proteins, factors that influence cell membrane properties, the module of HER2 expression, and agents with an impact on the progression of GC and peritoneal metastasis [10,11,12,13].

The most popular classification of GC is Laurén classification, which distinguishes between GC-diffuse and intestinal GC. These two types of GC are distinguished by their distinct characteristics, including their clinical features, morphology, genetics, and progression [14]. The intestinal type of GC primarily occurs in older patients and consists of tubular or glandular components with multiple stages of differentiation. The diffuse type of GC is comprised of weakly cohesive single cells without gland formation and mostly affects young patients; hereditary agents play the primary role in this type of GC [15].

The development of next-generation sequencing (NGS) has provided a high-throughput and systematic approach for discovering genetic alterations, mostly mutations in the cancer genome. Although some driver genes have been found, the molecular background of GC is still not completely understood. NGS studies have discovered several novel driver mutations with well-known driver genes in GC development [16,17,18]. Gene mutations that either predispose individuals to or induce GC can be organized based on different biological pathways, such as cell adhesion, genome integrity, chromatin remodeling, the *Wnt* pathway, the *RAS* family, the *MAPK* pathway, *RTKs*, or the *PIK* pathway [19].

The molecular classification of GC based on NGS data was recently established in a publication by Cristescu et al. (2015) [17], where gene expression data of 300 primary gastric tumors was explored. Four molecular subtypes of GC were selected: MSS/TP53^+^, MSS/TP53^−^, MSI, and MSS/EMT subtypes. They were assigned to different patterns of molecular alterations, the prognosis of GC patients, progression, and cancer prognosis. Currently, it is crucial to expand knowledge about the molecular classification of GC, which is mostly based on NGS data, as this might be a valuable approach for developing targeted therapy suitable for particular patients. The molecular profiling of GC is not only of value for uncovering the molecular basis of the disease, but also for identifying genes of clinical utility for therapeutic targets for GC.

In this study, an attempt was made to compare age-dependent genotypic and phenotypic characteristics of GC subtypes, with mutation profiling for the most frequent alterations in GC development, using high-throughput sequencing of both EOGC and CGC subtypes.

## 2. Materials and Methods

### 2.1. Study Group

Our study materials included samples from patients with CGCs diagnosed in years 1993–2003 and samples of patients with EOGCs, which were collected from the Academic Medical Centre in Amsterdam, as well as other Institutions located in The Netherlands, Finland, and Poland. Tumor samples were classified according to Laurén classification [14] as either intestinal or diffuse gastric adenocarcinomas. The study material was used in a previous study [5], in which immunohistochemical labeling was conducted to look for different expression patterns of several GC markers in EOGCs compared to CGCs. We selected 53 GC patients for the sequencing analysis—35 with EOGC and 18 with CGC. EOGC cases were classified as diffuse, with the age range of patients being between 21 and 45 years. CGC samples included intestinal histology and the age range of cases was 47–86 years. The clinical data for each GC patient is available in Table S1. The *CDH1* mutation status is described in Table S2, where the prediction for variant pathogenicity is presented. The group exhibited no pathogenic mutations in the *CDH1* gene, which, according to current knowledge, is consistent with HDGC. The study was approved by the Medical University of Lublin Bioethical Committee (Opinion no. KE-0254/322/2019.) and was conducted in accordance with the Declaration of Helsinki.

#### DNA Extraction

The tissues were collected as previously described by Sitarz et al. (2008) [20]. They were stored in liquid nitrogen. The genomic DNA extraction from formalin-fixed paraffin-embedded (FFPE) tissues was performed using the QIAamp DNA Mini kit (Qiagen, Venlo, the Netherlands) or the Puregene DNA Isolation kit (Gentra, MN, USA), in accordance with the manufacturer’s instructions. Isolated DNA was kept at −80 °C. DNA quantity assessment was conducted with the Quantus™ Fluorometer with the QuantiFluor^®^ dsDNA System, according to the protocol (Promega, Madison, WI, USA). The DNA quality was checked using the Agilent 2200 TapeStation and Genomic DNA ScreenTape (Agilent Technologies, Santa Clara, CA, USA). DNA samples were tested for high, middle, and low integrity. The DNA Integrity Number (DIN) algorithm was used to assess nucleic acid fragmentation. We selected samples with a DIN of at least 5 (where the scale is 1–10) for qPCR assessment. Further evaluation of the usefulness of the samples for sequencing was done with the Infinium HD FFPE QC Assay Protocol (Illumina, San Diego, CA, USA). Real-Time PCR by the CFX96 Touch^TM^ Real-Time PCR Detection System (Bio-Rad, Hercules, CA, USA) was performed to check the amplification status in each sample. The average quantification cycle (Cq) values for the quality control template_standard (QCT_ST) were subtracted from the average Cq value for each sample to compute the Delta Cq value for each sample. All samples with a Delta Cq value below 5 were selected for library preparation.

### 2.2. Library Preparation for Next-Generation Sequencing

Libraries for sequencing experiments were prepared using the Trusight Rapid Capture Preparation Kit (Illumina, San Diego, CA, USA), according to the manufacturer’s protocol. DNA samples were standardized to 50 ng and underwent a tagmentation step. The libraries generated from input genomic DNA were amplified and adapter-tagged multiple short library fragments of 220–350 base pairs long. The libraries for each sample were combined to obtain proper complexity for a single run. Biotin-labeled probes were used to target regions of interest. Further steps of hybridization were conducted with custom-synthesized oligos that captured genes with the assigned predisposition towards multiple cancer types. The sequencing panel, the TruSight Cancer Panel (Illumina, San Diego, CA, USA), targeted 94 genes and 284 single nucleotide polymorphisms (SNPs) linked to common and rare cancers. The complete list of target genes and SNPs is available on the website of Illumina. Standard pools were enriched with regions of interest through streptavidin-coated beads that bound to biotinylated probes. Then, DNA fragments were eluted from the beads and another round of hybridization and enrichment was performed. Final amplified post-capture library concentrations were assessed with the Quantus™ Fluorometer, according to the manufacturer’s protocol. Libraries were quantified by qPCR using the KAPA Library Quantification kit (KAPA Biosystems, Boston, MA, USA). The size of the obtained library fragments was evaluated with the Agilent 2200 TapeStation and High Sensitivity D1000 ScreenTape System (Agilent Technologies, Santa Clara, CA, USA). Post-capture enriched libraries were sequenced on the MiSeq Sequencing Platform (Illumina, San Diego, CA, USA) with the manufacturer’s workflow. The concentration of loaded libraries amounted to 10 pM. The sequencing experiment was performed with the MiSeq Reagent Kit v2 (300 cycles).

### 2.3. Data Processing

Results from each sample were mapped to the human reference genome GRCh37, also known as Human Genome version 19 (hg19), using the Burrows–Wheeler Aligner (BWA-mem, version 0.7.5). Readings with a low mapping quality score, unmapped readings, and duplicates were filtered out with Samtools (version 0.1.19) [21]. The local realignment of readings around indels (insertion or deletion) and detection of systematic errors in base quality scores were performed with the Genome Analysis Toolkit (GATK) [22]. Readings mapped outside the target region were removed. Variant calling for germline SNPs and indels was performed with the GATK HaplotypeCaller tool [23]. The callsets of SNPs and small insertions and deletions were separated for further filtering. The hard filters applied to variant callsets were, for SNPs, QD < 2.0, MQ < 40.0, FS > 60.0, HaplotypeScore > 13.0, QRankSum < −12.5, and ReadPosRankSum < −8.0, and for INDELs, QD < 2.0, ReadPosRankSum < −20.0, and FS > 200.0. Filtered variants were concatenated into one record (VCF file) and the discovered variants were then annotated with SnpEff (version 4.2) using GEMINI (GEnome MINIng 0.18.3) and loaded into the SQLite database [24].

### 2.4. In Silico Estimation of the Detected Variants

We attempted to provide an appropriate explanation for variants, which were prioritized as statistically significant for the age-dependent diversification of GC. There are several online annotations that provide variant interpretation, such as CADD and DANN scoring, FATHMM-XF, PROVEAN, and SIFT predictions, which are useful tools for estimating variants with a potentially high risk of GC development. Nevertheless, they also have several limitations. Firstly, factors of annotations differ in various aspects, from constitutions to functions. Secondly, each tool has a unique metric, which is hard to compare with the others. Thirdly, combined annotations might only deliver overlapping importance. In our study, we used the CADD scoring system, which is a framework employed for estimating the relative pathogenicity of human genetic variants, by incorporating multiple, different annotations into a single quantitative score.

## 3. Age-Dependent Genotypic and Phenotypic Characteristics of Gastric Cancer Subtypes

In the study cohort of 53 patients with GC, which included 18 cases with CGC and 35 with EOGC, we identified seven candidate genes for discriminating these two age-associated subtypes of GC (Table 1). Among them, we detected nine variants, which passed our selection criteria, including one splice_acceptor, four synonymous, and four missense variants. The frequency of each variant is presented in Table 1, separating EOGC and CGC groups, including the genotype, gene and chromosome location, type of alteration, and *p*-value (indicating the variant diversification among groups).

Three of the described variants were assigned to patients with the EOGC subtype. The missense variant (rs1799939) was found in the *RET* gene. The alteration frequency distribution among groups was, respectively, 46.2% of cases heterozygous for this variant in EOGC, 3.8% of EOGC cases homozygous for this variant, and 5.9% of CGC heterozygous for this variant. Interestingly, the homozygous missense variant (rs2959656) in the *Menin 1* gene (*MEN1*) was observed in 100% of the EOGCs and 82.4% of CGC cases. The heterozygous variant was very rare, presenting in 5.9% of CGC subtypes. The synonymous variant rs55986963 was only detected in *KIT* Proto-Oncogene Receptor Kinase (*KIT*) in the heterozygous form in 20.6% of EOGC cases. In CGC cases, the frequency of the homozygous variant was 5.6%.

### In Silico Estimation

In our study, we used different scoring and prediction systems for the relative assessment of the variant pathogenicity of a particular variant. The results are shown in Table 2.

We defined variants with C-Score ≥10 (top 10% in the ranking for pathogenicity) as 10% of the most deleterious substitutions. This criterion was fulfilled by the following four variants: rs1800862, rs10138997, rs2230009, and rs2959656. In practice, we cannot define pathogenicity as “deleterious” by only employing the C-score, as functional and/or clinical evidence is mandatory to confirm pathogenicity. The gnomAD database displayed variants with different frequencies among populations. The variants with CADD score higher than 10 were distributed respectively: rs1800862 (MAF = 0.04899), rs10138997 (MAF = 0.05851), rs2230009 (MAF = 0.05857), and rs2959656 (MAF = 0.9960).

## 4. High-Throughput Mutation Profiling Identifies the Most Frequent Mutations in EOGC and CGC Samples

**Figure 1:**
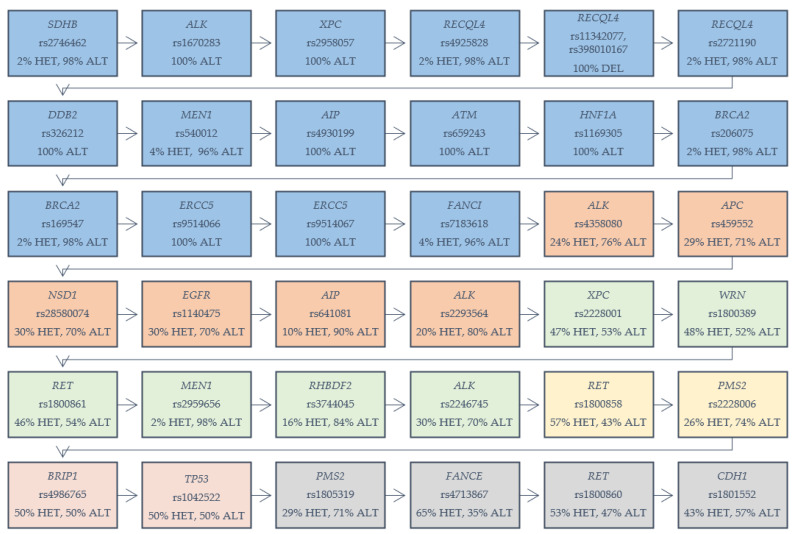
Variants in genes detected in gastric cancer samples (both early-onset gastric cancer (EOGC) and conventional gastric carcinoma (CGC)). HET: heterozygous variant; ALT: alternative homozygous variant. Five colored boxes are displayed: blue: 100% percent of cases with the mentioned variant; orange: one reference homozygote in the analyzed cases; green: two reference homozygotes; yellow: three reference homozygotes; red: four reference homozygotes; gray: five reference homozygotes.

In our cohort of 53 patients with GC, we identified 36 different variants, among 24 various genes, which are presented in Figure 1. Our selection criterion was the frequency of chosen variants among the analyzed group (both EOGC and CGC). The variants were selected based on the number of homozygous reference variants in analyzed cases. We determined the range of 0–5 numbers of homozygous reference variants in analyzed cases. We found 16 different variants among genes which were present in 100% of the total cohort, as heterozygous or alternative homozygous variants: *SDHB* (rs2746462), *ALK* (rs1670283), *XPC* (rs2958057), *RECQL4* (rs4925828; rs11342077, rs398010167; rs2721190), *DDB2* (rs326212), *MEN1* (rs540012), *AIP* (rs4930199), *ATM* (rs659243), *HNF1A* (rs1169305), *BRCA2* (rs206075; rs169547), *ERCC5* (rs9514066; rs9514067), and *FANCI* (rs7183618). Six variants with one or two homozygous reference variants were detected in several genes: *ALK* (rs4358080; rs2293564)*, APC* (rs459552)*, NSD1* (rs28580074)*, EGFR* (rs1140475)*, AIP* (rs641081) and *XPC* (rs2228001), *WRN* (rs1800389), *RET* (rs1800861), *MEN1* (rs2959656), *RHBDF2* (rs3744045), and *ALK* (rs2246745). Two variants in genes—*RET* (rs1800858) and *PMS2* (rs2228006) were present among the samples, with three homozygous reference variants. Two variants were detected with four references-in genes *BRIP1* (rs4986765) and *TP53* (rs1042522). Genes *PMS2* (rs1805319), *FANCE* (rs4713867), *RET* (rs1800860), and *CDH1* (rs1801552)—each with a variant showing five references among the analyzed samples.

Among the thirty-six variants identified in our patient cohort, it is expected that sixteen of them (fifteen missense and one frameshift) will result in amino acid substitution (Table 3). Nine variants according to the CADD scoring system, with C-Score ≥ 10, were described as 10% of the most deleterious substitutions and thus most likely affect gene or protein function. Twenty of the detected variants were synonymous substitutions in which the produced amino acid sequence was not modified.

Nine of the mentioned variants with C-Score ≥ 10 were sequences of nine different genes: *AIP, BRCA2, ERCC5, APC, XPC, RET, MEN1, RHBDF2,* and *PMS2*. These genes are known to have roles in different processes and syndromes, such as DNA damage responses, DNA repair, multiple endocrine neoplasia type 1 syndrome, the aryl hydrocarbon receptor pathway, the *Wnt* signaling pathway, and *ERK* signaling. All of the variants are listed in Table 3.

## 5. Discussion

**Figure 2:**
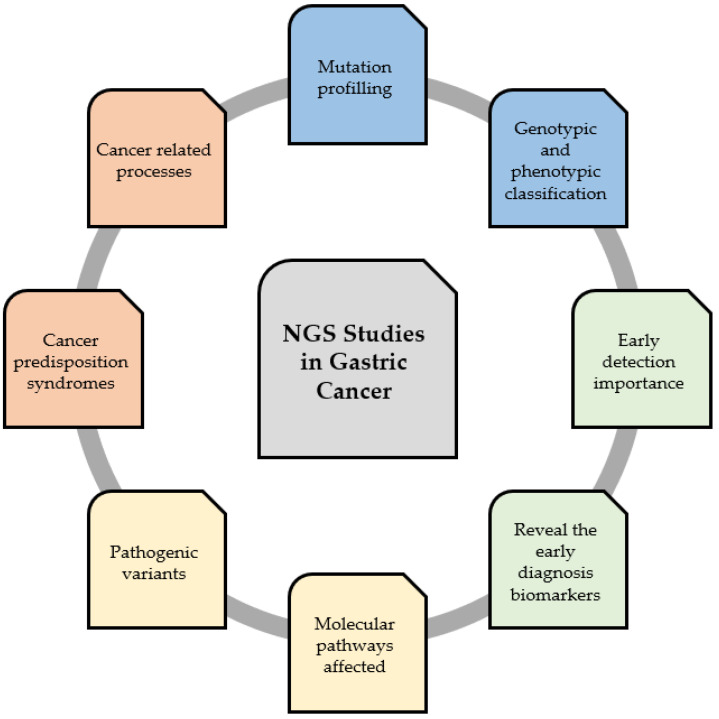
Next-generation sequencing (NGS) as a useful tool for studying the development of gastric carcinoma in a high-throughput way.

GC is one of the most common cancers and one of the most frequent causes of cancer-related deaths [25]. The prognosis and 5-year survival rate of patients with GC are still very poor. NGS is a useful tool for revealing the mutation profiling, importance of early and late development, and genotypic and phenotypic classification of GC. This technology helps to identify specific biomarkers, tumor suppressor genes, and carcinogens, as well as facilitate the understanding of mechanisms and affected pathways of GC tumorigenesis [26]. Therefore, this might be applied in the future for early diagnosis or personal treatment by either determining the particular GC biomarker or a specific drug-resistant gene [27]. Figure 2 summarizes the conclusions that can be drawn from the obtained results in the context of applying NGS studies as a useful tool for studying GC development in a high-throughput way.

We used the high-throughput sequencing of GC patients to compare and characterize age-dependent genotypic and phenotypic characteristics of GC subtypes. Based on the results obtained in this study, we identified potential candidate genes for distinguishing between EOGC and CGC. We were able to identify seven candidate genes, as well as nine variants, among them, which were statistically significantly different in these two subgroups. Variants, including rs1799939 (CADD = 8.26, MAF = 0.1847, *p* = 0.010), rs2959656 (CADD = 11.00, MAF = 0.9960, *p* = 0.041), and rs55986963 (CADD = 7.36, MAF = 0.03027, *p* = 0.052), were predominantly detected in EOGC patients. Interestingly, variant rs2959656 was found in 100% of cases of EOGC as homozygous. According to the NCBI ClinVar database, variant rs1799939 in the *RET* gene is associated with multiple endocrine neoplasia, hereditary cancer-predisposing syndrome, renal dysplasia, and pheochromocytoma. Activation of the RET proto-oncogene might constitute one of the molecular drivers of gastric inflammatory and neoplastic diseases [3]. Variant rs2959656 was detected in the *MEN1* gene. Multiple Endocrine Neoplasia type 1 (MEN1) is a rare hereditary endocrine cancer syndrome, mainly leading to tumors of the parathyroid glands, endocrine gastroenteropancreatic tract, and the anterior pituitary [28]. Other endocrine and non-endocrine neoplasms are also observed, including GC [29]. Variant rs55986963 in the *KIT* gene, according to the NCBI ClinVar database, is associated with gastrointestinal stromal tumors (GISTs), mastocytosis, and partial albinism. Capelli et al. (2016) [30] also investigated exons 9, 11, 13, and 17 of the *KIT* gene by direct sequencing and showed that *KIT* mutations were observed in 53.8% of patients with gastric GISTs. Besides, *KIT* deletions in exon 11, mostly those involving codons 557, 558, and 559, were primarily associated with the more aggressive gastric GIST phenotype and a higher probability of death or relapse.

Variants that were most frequently detected among CGC included long deletion in the *STK11* gene (*p* = 0.004), rs1800862 (CADD = 10.26, MAF = 0.04899, *p* = 0.006), rs10138997 (CADD = 14.99, MAF = 0.05851, *p* = 0.009), rs3810812 (CADD = 1.21, MAF = 0.5139, *p* = 0.018), rs2230009 (CADD = 13.29, MAF = 0.05857, *p* = 0.024), and rs28516461 (CADD = 0.14, MAF = 0.01392, *p* = 0.054). Variant rs1800862 (*RET*), according to NCBI ClinVar, is associated with multiple endocrine neoplasia, pheochromocytoma, and Hirschsprung disease, and is dominant. Variant rs10138997 (*FANCM*) and variants rs3810812 and rs28516461 (*SLX4*) are Fanconi anemia (FA) disease associated (NCBI ClinVar). The study performed by Swift et al. showed 102 deaths in relatives of eight FA families and a higher rate of leukemia and gastric, colorectal, and tongue cancer [31]. The variant rs2230009 (*WRN*) is associated with Werner syndrome (NCBI ClinVar). Patients with Werner syndrome present with an increased incidence of cancer, indicating that the lack of a proper *WRN* function affects tumorigenesis [32].

Our study of a cohort of 53 patients with GC allowed us to identify 36 different variants, among 24 various genes, which turned out to be the most probable genetic alterations, causing the development of GC. Importantly, we displayed 16 different variants that were detected in 100% of the total cohort. DNA damage appears to be the underlying cause of cancer [33] and deficiencies in DNA repair genes induce the development of different types of cancer [34]. If DNA repair is defective, DNA damage accumulates, increasing the number of mutations that occur due to error-prone translational synthesis, which consequently leads to the initiation of cancer development. Alterations in DNA double-strand break repair and DNA damage-response genes were detected in our study, such as variants rs2958057 (*XPC*); rs4925828, rs11342077, rs398010167 and rs2721190 (*RECQL4);* rs206075 and rs169547 (*BRCA2);* rs659243 (*ATM*); rs326212 (*DDB2*); rs9514066 and rs9514067 (*ERCC5)*. It has been shown in different studies that the response to DNA damage plays an important role in the pathobiology of GCs [35,36,37]. The FA pathway constitutes a part of the DNA-damage network, including breast cancer-susceptibility proteins BRCA1 and BRCA2. The pathway is activated by ataxia telangiectasia and Rad3-related (ATR) kinase; however, the underlying mechanism remains unclear. A new study has demonstrated that the major switch activating the pathway is the ATR-dependent phosphorylation of FANCI [38]. Variant rs2746462 (*SDHB*) is related to paraganglioma and gastric stromal cell sarcoma, as well as the hereditary cancer-predisposing syndrome. Miettinen and Lasota (2014) described that approximately half of the patients with GISTs present *SDH* subunit gene mutations, mostly germline, with both alleles being inactivated in the tumor cells [39]. Variant rs1670283 (*ALK*) is associated with the hereditary cancer-predisposing syndrome and neuroblastoma 3 (NCBI ClinVar). Variant rs1169305 (*HNF1A*) is assigned to maturity-onset diabetes of young type 3 (MODY3) and might also result in hepatic adenomas (NCBI ClinVar). Variant rs4930199 (*AIP*) is associated with the hereditary cancer-predisposing syndrome and familial isolated pituitary adenomas.

Six of the variants with one and two homozygous reference variants were displayed in genes: *ALK*, *APC*, *NSD1*, *EGFR*, *AIP* and *XPC*, *WRN*, *RET*, *MEN1*, *RHBDF2*, and *ALK.* Two variants in genes—*RET* and *PMS2*—were detected with three homozygous reference variants. For *BRIP1* and *TP53* genes, two variants were detected with four references. Genes *PMS2*, *FANCE*, *RET*, and *CDH1*—each with variants showing five references among the analyzed group. Variant rs459552 (*APC*) is associated with familial adenomatous polyposis 1 and hereditary cancer-predisposing syndrome. The APC mutation is involved in the carcinogenesis of the intestinal type of GC and related to the LOH pathway in GC [40]. Variant rs28580074 (*NSD1*) is prevalent in Weaver syndrome and Sotos syndrome according to NCBI ClinVar. Variant rs1140475 is present in *EGFR*. *EGFR, HER2,* and *MET* signaling is important, especially in proximal non-diffuse tumors, and constitutes the logical targets for molecular therapy [41]. Variant rs3744045 in the *RHBDF2* gene is associated with Howel–Evans syndrome. *RHBDF2* seems to regulate oncogenic and non-canonical *TGFB1* signaling. GC-associated fibroblasts increase their motility, via the expression of rhomboid 5 homolog 2, and the ability to induce the invasiveness of GC cells [42]. Variant rs2228006 (*PMS2)* is common for hereditary nonpolyposis colorectal cancer type 4 and mismatch repair cancer syndrome. *BRIP1* and variant rs4986765 are related to familial cancer of the breast, neoplasms of the ovary, hereditary cancer-predisposing syndrome, and Fanconi anemia, which is also associated with the variant rs4713867 (*FANCE*) gene. The *TP53* alteration (rs1042522) is related to Li–Fraumeni syndrome and hereditary cancer-predisposing syndrome. Besides, p53 alterations are displayed quite early in the development of GC, even being present in the non-neoplastic mucosa, and their incidence increases with the progression of GC [43]. Even though our study was limited due to a low number of samples, it provides a significant insight into genetic alterations that occur in GC, primarily EOGC and CGC. Further studies with larger groups of patients are needed, since the differences between EOGC and CGC cases may possibly be more significant.

## 6. Conclusions

We were able to compare the age-dependent genotypic and phenotypic profile of GC patients, focusing on the most frequent alterations in GC development, both EOGC and CGC, using high-throughput sequencing technology. We found variants among several genes, which might be considered for future studies on the early detection and diagnosis of GC. We displayed the processes and syndromes involved in EOGC and CGC development. Mutation prediction tools enable us to estimate potential candidate biomarkers with a pathogenic impact on disease development. This study primarily placed emphasis on EOGC development, which is less exposed to environmental factors and constitutes a good model for further studies on the genetic background of GC development.

## Figures and Tables

**Table 1:** Candidate genes employed for age-dependent classification of gastric carcinoma.

Phenotype	Genotype	Reference	Frequency	*p* Value	Chr	Gene	Variant Type	dbSNP
CGC	a *	b *	0.471	0.004	19	*STK11*	Splice_acceptor	NA
EOGC	a *	b *	0.067					
CGC	C/T	C	0.313	0.006	10	*RET*	Synonymous	rs1800862
CGC	T/T	C	0.063					
EOGC	C/T	C	0.031					
EOGC	T/T	C	0.000					
CGC	C/T	C	0.389	0.009	14	*FANCM*	Missense	rs10138997
EOGC	C/T	C	0.059					
CGC	G/A	G	0.059	0.010	10	*RET*	Missense	rs1799939
CGC	A/A	G	0.000					
EOGC	G/A	G	0.462					
EOGC	A/A	G	0.038					
CGC	A/G	A	0.353	0.018	16	*SLX4*	Synonymous	rs3810812
CGC	G/G	A	0.588					
EOGC	A/G	A	0.324					
EOGC	G/G	A	0.265					
CGC	G/A	G	0.278	0.024	8	*WRN*	Missense	rs2230009
EOGC	G/A	G	0.029					
CGC	T/C	T	0.059	0.041	11	*MEN1*	Missense	rs2959656
CGC	C/C	T	0.824					
EOGC	T/C	T	0.000					
EOGC	C/C	T	1.000					
CGC	A/G	A	0.000	0.052	4	*KIT*	Synonymous	rs55986963
CGC	G/G	A	0.056					
EOGC	A/G	A	0.206					
EOGC	G/G	A	0.000					
CGC	G/A	G	0.222	0.054	16	*SLX4*	Synonymous	rs28516461
CGC	A/A	G	0.000					
EOGC	G/A	G	0.029					
EOGC	A/A	G	0.059					

NA: not applicable. a * Sequence with deletion: GAGGTAGGCACGTGCTAGGGGGGGCCCTGGGGCGCCCCCTCCCGGGCACTCCCTGAGGGCTGCACGGCACCGCCAC/G. b * Reference sequence: GAGGTAGGCACGTGCTAGGGGGGGCCCTGGGGCGCCCCCTCCCGGGCACTCCCTGAGGGCTGCACGGCACCGCCAC/GAGGTAGGCACGTGCTAGGGGGGGCCCTGGGGCGCCCCCTCCCGGGCACTCCCTGAGGGCTGCACGGCACCGCCAC.

**Table 2:** Relative assessment of the variant pathogenicity.

dbSNP	CADD Score	DANN Score	FATHMM-XF Prediction	SIFT Prediction	PROVEAN Prediction	gnomAD MAF (European Non-Finnish)
rs1800862	10.26	0.6808	Benign (high conf.)	Tolerated	Neutral	0.04899
rs10138997	14.99	0.9662	Benign	Tolerated	Neutral	0.05851
rs1799939	8.26	0.8595	Benign	Tolerated	Neutral	0.1847
rs3810812	1.21	0.3514	Benign (high conf.)	Tolerated	Neutral	0.5139
rs2230009	13.29	0.3339	Benign (high conf.)	Tolerated	Neutral	0.05857
rs2959656	11.00	0.5414	Benign (high conf.)	Tolerated	Neutral	0.9960
rs55986963	7.36	0.4165	Benign (high conf.)	Tolerated	Neutral	0.03027
rs28516461	0.14	0.8924	Benign (high conf.)	Tolerated	Neutral	0.01392

**Table 3:** In silico estimation of the variant’s pathogenicity. NA: not applicable.

dbSNP	Variant Type	Chr	Gene	CADD Score (Scaled)	DANN Score	FATHMM-XF Prediction	SIFT Prediction	PROVEAN Prediction	gnomAD MAF (European Non-Finnish)
rs2746462	Synonymous	1	*SDHB*	6.608	0.6939	Benign (high conf.)	Tolerated	Neutral	0.9724
rs1670283	Missense	2	*ALK*	0.648	0.5289	Benign (high conf.)	Tolerated	Neutral	0.9998
rs2958057	Synonymous	3	*XPC*	6.712	0.7056	Benign (high conf.)	Tolerated	Neutral	1.000
rs4925828	Synonymous	8	*RECQL4*	5.112	0.6599	Benign (high conf.)	Tolerated	NA	0.9994
rs11342077, rs398010167	Frameshift	8	*RECQL4*	NA	NA	NA	NA	NA	NA
rs2721190	Missense	8	*RECQL4*	5.653	0.4532	Benign	Tolerated	NA	0.9993
rs326212	Synonymous	11	*DDB2*	9.276	0.5475	Benign (high conf.)	Tolerated	Neutral	1.000
rs540012	Synonymous	11	*MEN1*	8.171	0.5409	Benign (high conf.)	Tolerated	Neutral	0.9999
rs4930199	Missense	11	*AIP*	15.65	0.7273	Benign	Tolerated	Neutral	1.000
rs659243	Missense	11	*ATM*	7.875	0.7855	Benign (high conf.)	Tolerated	Neutral	1.000
rs1169305	Missense	12	*HNF1A*	9.746	0.7163	Benign	Tolerated	Neutral	0.9998
rs206075	Synonymous	13	*BRCA2*	2.651	0.4723	Benign (high conf.)	Tolerated	Neutral	0.9996
rs169547	Missense	13	*BRCA2*	11.56	0.1694	Benign (high conf.)	Tolerated	Neutral	0.9997
rs9514066	Missense	13	*ERCC5*	21.0	0.9962	Benign	Tolerated, Damaging	Neutral	1.000
rs9514067	Missense	13	*ERCC5*	1.290	0.6273	Benign	Tolerated	Neutral	0.9999
rs7183618	Synonymous	15	*FANCI*	3.241	0.4015	Benign (high conf.)	Tolerated	Neutral	0.9469
rs4358080	Synonymous	2	*ALK*	8.136	0.5627	Benign (high conf.)	Tolerated	Neutral	0.9086
rs459552	Missense	5	*APC*	18.00	0.8086	Benign (high conf.)	Tolerated	Neutral	0.7677
rs28580074	Synonymous	5	*NSD1*	5.986	0.7262	Benign (high conf.)	Tolerated	Neutral	0.8768
rs1140475	Synonymous	7	*EGFR*	6.939	0.4885	Benign (high conf.)	Tolerated	Neutral	0.8741
rs641081	Missense	11	*AIP*	4.743	0.6594	Benign	Tolerated	Neutral	0.9979
rs2293564	Synonymous	2	*ALK*	1.005	0.3863	Benign (high conf.)	Tolerated	Neutral	0.9187
rs2228001	Missense	3	*XPC*	17.09	0.9017	Benign	Tolerated	Neutral	0.5939
rs1800389	Synonymous	8	*WRN*	4.911	0.4329	Benign (high conf.)	Tolerated	Neutral	0.7031
rs1800861	Synonymous	10	*RET*	10.76	0.7308	Benign (high conf.)	Tolerated	Neutral	0.7668
rs2959656	Missense	11	*MEN1*	11.00	0.5414	Benign (high conf.)	Tolerated	Neutral	0.9960
rs3744045	Missense	17	*RHBDF2*	21.1	0.9951	Pathogenic	Tolerated, Damaging	Neutral	0.9366
rs2246745	Synonymous	2	*ALK*	4.814	0.5041	Benign (high conf.)	Tolerated	Neutral	0.8143
rs1800858	Synonymous	10	*RET*	1.167	0.3128	Benign (high conf.)	Tolerated	Neutral	0.7384
rs2228006	Missense	7	*PMS2*	10.11	0.2203	Benign (high conf.)	Tolerated	Neutral	0.8501
rs4986765	Synonymous	17	*BRIP1*	9.957	0.6153	Benign (high conf.)	Tolerated	Neutral	0.6603
rs1042522	Missense	17	*TP53*	9.176	0.5704	Benign	Tolerated	Neutral	0.7366
rs1805319	Synonymous	7	*PMS2*	0.168	0.7105	Benign (high conf.)	Tolerated	Neutral	0.8160
rs4713867	Synonymous	6	*FANCE*	1.716	0.3909	Benign (high conf.)	Tolerated	Neutral	0.6747
rs1800860	Synonymous	10	*RET*	6.248	0.3745	Benign (high conf.)	Tolerated	Neutral	0.6900
rs1801552	Synonymous	16	*CDH1*	5.661	0.4494	Benign (high conf.)	Tolerated	Neutral	0.6252

## Supplementary Materials

The following are available online at https://www.mdpi.com/2072-6694/12/7/1981/s1: Table S1: A table containing the clinical characteristics of the studied group, Table S2: A table with *in silico* estimation of the *CDH1* mutation status among GC patients.

## Abbreviations

CGC	conventional gastric carcinoma
EOGC	early-onset gastric cancer
FA	Fanconi anemia
FFPE	formalin-fixed paraffin-embedded
GC	gastric cancer
GIST	gastrointestinal stromal tumor
MEN1	multiple endocrine neoplasia type 1
NGS	next-generation sequencing
SNPs	single nucleotide polymorphisms

## References

[1] Gullo I., Carneiro F., Oliveira C., Almeida G.M. (2018). Heterogeneity in Gastric Cancer: From Pure Morphology to Molecular Classifications. Pathobiology.

[2] Gao J.P., Xu W., Liu W.T., Yan M., Zhu Z.G. (2018). Tumor heterogeneity of gastric cancer: From the perspective of tumor-initiating cell. World J. Gastroenterol..

[3] Zhang X.Y., Zhang P.Y. (2017). Gastric cancer: Somatic genetics as a guide to therapy. J. Med. Genet..

[4] Kokkola A., Sipponen P. (2001). Gastric carcinoma in young adults. Hepato Gastroenterol..

[5] Milne A.N., Carvalho R., Morsink F.M., Musler A.R., de Leng W.W., Ristimäki A., Offerhaus G.J. (2006). Early-onset gastric cancers have a different molecular expression profile than conventional gastric cancers. Mod. Pathol..

[6] Pucułek M., Machlowska J., Wierzbicki R., Baj J., Maciejewski R., Sitarz R. (2018). *Helicobacter pylori* associated factors in the development of gastric cancer with special reference to the early-onset subtype. Oncotarget.

[7] Rocco A., Nardone G. (2007). Diet, *H pylori* infection and gastric cancer: Evidence and controversies. World J. Gastroenterol..

[8] Baj J., Brzozowska K., Forma A., Maani A., Sitarz E., Portincasa P. (2020). Immunological Aspects of the Tumor Microenvironment and Epithelial-Mesenchymal Transition in Gastric Carcinogenesis. Int. J. Mol. Sci..

[9] Baj J., Korona-Głowniak I., Forma A., Maani A., Sitarz E., Rahnama-Hezavah M., Radzikowska E., Portincasa P. (2020). Mechanisms of the Epithelial–Mesenchymal Transition and Tumor Microenvironment in *Helicobacter pylori*-Induced Gastric Cancer. Cells.

[10] Machlowska J., Maciejewski R., Sitarz R. (2018). The Pattern of Signatures in Gastric Cancer Prognosis. Int. J. Mol. Sci..

[11] Machlowska J., Baj J., Sitarz M., Maciejewski R., Sitarz R. (2020). Gastric Cancer: Epidemiology, Risk Factors, Classification, Genomic Characteristics and Treatment Strategies. Int. J. Mol. Sci..

[12] Rimini M., Casadei-Gardini A., Ravaioli A., Rovesti G., Conti F., Borghi A., Dall’Aglio A.C., Bedogni G., Domenicali M., Giacomoni P. (2020). Could Inflammatory Indices and Metabolic Syndrome Predict the Risk of Cancer Development? Analysis from the Bagnacavallo Population Study. J. Clin. Med..

[13] Salati M., Pipitone S., Rimini M., Gelsomino F., Gardini A.C., Andrikou K., Schipilliti F., Cortesi G., Cassanelli L., Caffari E. (2019). Immune-inflammatory and clinicopathologic prognostic factors in a Western cohort of resected gastric cancers (GCs). Ann. Oncol..

[14] Laurén P. (1965). The two histological main types of gastric carcinoma: Diffuse and so-called intestinal-type carcinoma. An attempt at a histo-clinical classification. Acta Pathol. Microbiol. Scand..

[15] Bosman F.T., Carneiro F., Hruban R.H., Theise N.D. (2010). WHO Classification of Tumours of the Digestive System.

[16] Liang H., Kim Y.H. (2013). Identifying molecular drivers of gastric cancer through next-generation sequencing. Cancer Lett..

[17] Cristescu R., Lee J., Nebozhyn M., Kim K.M., Ting J.C., Wong S.S., Liu J., Yue Y.G., Wang J., Yu K. (2015). Molecular analysis of gastric cancer identifies subtypes associated with distinct clinical outcomes. Nat. Med..

[18] Cancer Genome Atlas Research Network (2014). Comprehensive molecular characterization of gastric adenocarcinoma. Nature.

[19] Riquelme I., Saavedra K., Espinoza J.A., Weber H., García P., Nervi B., Garrido M., Corvalán A.H., Roa J.C., Bizma C. (2015). Molecular classification of gastric cancer: Towards a pathway-driven targeted therapy. Oncotarget.

[20] Sitarz R., Leguit R.J., de Leng W.W., Polak M., Morsink F.M., Bakker O., Maciejewski R., Offerhaus G.J., Milne A.N. (2008). The COX-2 promoter polymorphism -765 G>C is associated with early-onset, conventional and stump gastric cancers. Mod. Pathol..

[21] Li H., Handsaker B., Wysoker A., Fennell T., Ruan J., Homer N., Marth G., Abecasis G., Durbin R. (2009). 1000 Genome Project Data Processing Subgroup. The Sequence Alignment/Map format and SAMtools. Bioinformatics.

[22] McKenna A., Hanna M., Banks E., Sivachenko A., Cibulskis K., Kernytsky A., Garimella K., Altshuler D., Gabriel S., Daly M. (2010). The Genome Analysis Toolkit: A MapReduce framework for analyzing next-generation DNA sequencing data. Genome Res..

[23] Narzisi G., O’Rawe J.A., Iossifov I., Fang H., Lee Y.H., Wang Z., Wu Y., Lyon G.J., Wigler M., Schatz M.C. (2014). Accurate *de novo* and transmitted indel detection in exome-capture data using microassembly. Nat. Methods.

[24] Cingolani P., Platts A., Wang le L., Coon M., Nguyen T., Wang L., Land S.J., Lu X., Ruden D.M. (2012). A program for annotating and predicting the effects of single nucleotide polymorphisms, SnpEff: SNPs in the genome of Drosophila melanogaster strain w1118; iso-2; iso-3. Fly.

[25] Patru C.L., Surlin V., Georgescu I., Patru E. (2013). Current issues in gastric cancer epidemiology. Rev. Med. Chir. Soc. Med. Nat. Iasi.

[26] Kamps R., Brandão R.D., Bosch B.J., Paulussen A.D., Xanthoulea S., Blok M.J., Romano A. (2017). Next-Generation Sequencing in Oncology: Genetic Diagnosis, Risk Prediction and Cancer Classification. Int. J. Mol. Sci..

[27] Shyr D., Liu Q. (2013). Next generation sequencing in cancer research and clinical application. Biol. Proced. Online.

[28] Marini F., Falchetti A., Luzi E., Tonelli F., Maria Luisa B., Riegert-Johnson D.L., Boardman L.A., Hefferon T., Roberts M. (2009). Multiple Endocrine Neoplasia Type 1 (MEN1) Syndrome. Cancer Syndromes.

[29] Debelenko L.V., Emmert-Buck M.R., Zhuang Z., Epshteyn E., Moskaluk C.A., Jensen R.T., Liotta L.A., Lubensky I.A. (1997). The multiple endocrine neoplasia type I gene locus is involved in the pathogenesis of type II gastric carcinoids. Gastroenterology.

[30] Capelli L., Petracci E., Quagliuolo V., Saragoni L., Colombo P., Morgagni P., Calistri D., Tomezzoli A., Di Cosmo M., Roviello F. (2016). GISTs: Analysis of *c-Kit*, *PDGFRA* and *BRAF* mutations in relation to prognosis and clinical pathological characteristics of patients—A GIRCG study. Eur. J. Surg. Oncol..

[31] Swift M. (1971). Fanconi’s anaemia in the genetics of neoplasia. Nature.

[32] Goto M., Miller R.W., Ishikawa Y., Sugano H. (1996). Excess of rare cancers in Werner syndrome (adult progeria). Cancer Epidemiol. Prev. Biomark..

[33] Kastan M.B. (2008). DNA damage responses: Mechanisms and roles in human disease: 2007 G.H.A. Clowes Memorial Award Lecture. Mol. Cancer Res..

[34] Harper J.W., Elledge S.J. (2007). The DNA damage response: Ten years after. Mol. Cell..

[35] Arai H., Wada R., Ishino K., Kudo M., Uchida E., Naito Z. (2018). Expression of DNA damage response proteins in gastric cancer: Comprehensive protein profiling and histological analysis. Int. J. Oncol..

[36] Ronchetti L., Melucci E., De Nicola F., Goeman F., Casini B., Sperati F., Pallocca M., Terrenato I., Pizzuti L., Vici P. (2017). DNA damage repair and survival outcomes in advanced gastric cancer patients treated with first-line chemotherapy. Int. J. Cancer..

[37] Lee H.E., Han N., Kim M.A., Lee H.S., Yang H.K., Lee B.L., Kim W.H. (2014). DNA damage response-related proteins in gastric cancer: ATM, Chk2 and p53 expression and their prognostic value. Pathobiology.

[38] Wang W. (2008). A major switch for the Fanconianemia DNA damage-response pathway. Nat. Struct. Mol. Biol..

[39] Miettinen M., Lasota J. (2014). Succinate dehydrogenase deficient gastrointestinal stromal tumors (GISTs)—A review. Int. J. Biochem. Cell Biol..

[40] Fang D.C., Luo Y.H., Yang S.M., Li X.A., Ling X.L., Fang L. (2002). Mutation analysis of APC gene in gastric cancer with microsatellite instability. World J. Gastroenterol..

[41] Wong H., Yau T. (2013). Molecular targeted therapies in advanced gastric cancer: Does tumor histologymatter. Therap. Adv. Gastroenterol..

[42] Ishimoto T., Miyake K., Nandi T., Yashiro M., Onishi N., Huang K.K., Lin S.J., Kalpana R., Tay S.T., Suzuki Y. (2017). Activation of Transforming Growth Factor Beta 1 Signaling in Gastric Cancer-associated Fibroblasts Increases Their Motility, via Expression of Rhomboid 5 Homolog 2, and Ability to Induce Invasiveness of Gastric Cancer Cells. Gastroenterology.

[43] Fenoglio-Preiser C.M., Wang J., Stemmermann G.N., Noffsinger A. (2003). TP53 and gastric carcinoma: A review. Hum. Mutat..

