# Supplementary material for: High-Throughput Sequencing of Gastric Cancer Patients: Unravelling Genetic Predispositions Towards an Early-Onset Subtype
Source: Cancers (Basel). 2020 Jul 21;12(7):1981. doi: 10.3390/cancers12071981 (PMC7409326; doi:10.3390/cancers12071981)
Supplement: Supplementary file 1 [file cancers-12-01981-s001.pdf]

cancers

High-Throughput Sequencing of Gastric Cancer Patients: Unravelling Genetic Predispositions Towards an Early-Onset Subtype

Julita Machlowska, Przemysław Kapusta, Jacek Baj, Folkert H. M. Morsink, Paweł Wołkow, Ryszard Maciejewski, G. Johan A. Offerhaus and Robert Sitarz

Table S1. Clinical characteristics of the studied group.

Patient ID	Age of Onset	Age-Dependent Subtype	Histology	Family History	*CDH1* Mutation Status (dbSNP)	Treatment Strategy
**1**	>45	CGC	Intestinal	Negative	rs1801552, rs9929218	Surgical resection
**2**	>45	CGC	Intestinal	Negative	rs1801552	Surgical resection
**5**	>45	CGC	Intestinal	Negative	rs1801552	Surgical resection
**7**	>45	CGC	Intestinal	Negative	rs1801552	Surgical resection
**10**	>45	CGC	Intestinal	Negative	rs1801552	Surgical resection
**17**	>45	CGC	Intestinal	Negative	rs33969373, rs1801552, rs2229044, rs9929218	Surgical resection
**21**	>45	CGC	Intestinal	Negative	rs1801552	Surgical resection
**22**	>45	CGC	Intestinal	Negative	rs1801552, rs33964119	Surgical resection
**26**	>45	CGC	Intestinal	Negative	rs1801552, rs9929218	Surgical resection
**31**	>45	CGC	Intestinal	Negative	rs1801552, rs9929218	Surgical resection
**35**	>45	CGC	Intestinal	Negative	rs1801552, rs9929218	Surgical resection
**37**	>45	CGC	Intestinal	Negative	rs35741240, rs1801552	Surgical resection
**45**	>45	CGC	Intestinal	Negative	rs1801552, rs9929218	Surgical resection
**63N**	>45	CGC	Intestinal	Negative	rs1801552, rs9929218	Surgical resection
**72**	>45	CGC	Intestinal	Negative	rs1801552	Surgical resection
**82**	>45	CGC	Intestinal	Negative	rs1801552	Surgical resection
**84**	>45	CGC	Intestinal	Negative	rs1801552	Surgical resection
**91N**	>45	CGC	Diffuse	Negative	rs1801552	Surgical resection
**Y3**	≤45	EOGC	Diffuse	Negative	rs1801552	Surgical resection
**Y68**	≤45	EOGC	Diffuse	Negative	rs9929218	Surgical resection
**Y77**	≤45	EOGC	Diffuse	Negative	rs1801552, rs9929218	Surgical resection
**Y88**	≤45	EOGC	Diffuse	Negative	rs761309816	Surgical resection
**Y102**	≤45	EOGC	Diffuse	Negative	rs1801552	Surgical resection
**Y103**	≤45	EOGC	Diffuse	Negative	rs1801552, rs9929218	Surgical resection

**Y104**	≤45	EOGC	Diffuse	Negative	rs761309816, rs1801552, rs61747636, rs9929218	Surgical resection
**Y105**	≤45	EOGC	Diffuse	Negative	Negative	Surgical resection
**Y109B**	≤45	EOGC	Diffuse	Negative	rs1801552	Surgical resection
**Y110B**	≤45	EOGC	Diffuse	Negative	rs1801552	Surgical resection
**Y115B**	≤45	EOGC	Diffuse	Negative	rs1801552	Surgical resection
**Y123B**	≤45	EOGC	Diffuse	Negative	rs1801552, rs9929218	Surgical resection
**Y128B**	≤45	EOGC	Diffuse	Negative	rs1801552	Surgical resection
**Y132B**	≤45	EOGC	Diffuse	Negative	rs1801023, rs9929218	Surgical resection
**Y134B**	≤45	EOGC	Diffuse	Negative	rs1801552, rs9929218	Surgical resection
**Y135**	≤45	EOGC	Diffuse	Negative	rs1801552	Surgical resection
**Y137B**	≤45	EOGC	Diffuse	Negative	Negative	Surgical resection
**Y143C**	≤45	EOGC	Diffuse	Negative	rs1801552, rs9929218	Surgical resection
**Y144C**	≤45	EOGC	Diffuse	Negative	rs1801552	Surgical resection
**Y145B**	≤45	EOGC	Diffuse	Negative	rs1801552, rs9929218	Surgical resection
**Y146B**	≤45	EOGC	Diffuse	Negative	rs1801025, rs1801552	Surgical resection
**Y147C**	≤45	EOGC	Diffuse	Negative	rs1801552	Surgical resection
**Y148C**	≤45	EOGC	Diffuse	Negative	Negative	Surgical resection
**Y151B**	≤45	EOGC	Diffuse	Negative	rs1801552, rs9929218	Surgical resection
**Y159C**	≤45	EOGC	Diffuse	Negative	rs1801552	Surgical resection
**Y161C**	≤45	EOGC	Diffuse	Negative	rs1801552, rs9929218	Surgical resection
**Y162C**	≤45	EOGC	Diffuse	Negative	rs1801552, rs9929218	Surgical resection
**Y165B**	≤45	EOGC	Diffuse	Negative	Negative	Surgical resection
**Y167B**	≤45	EOGC	Diffuse	Negative	rs1801552	Surgical resection
**Y168C**	≤45	EOGC	Diffuse	Negative	rs1801552	Surgical resection
**Y169C**	≤45	EOGC	Diffuse	Negative	rs1801552, rs9929218	Surgical resection
**Y179F**	≤45	EOGC	Diffuse	Negative	rs1801552, rs9929218	Surgical resection
**Y183B**	≤45	EOGC	Diffuse	Negative	rs1801552, rs9929218	Surgical resection
**Y188B**	≤45	EOGC	Diffuse	Negative	rs1801552, rs9929218	Surgical resection
**Y199B**	≤45	EOGC	Diffuse	Negative	rs35741240, rs1801552	Surgical resection

Table S2. *In silico* estimation of *CDH1* mutations status among GC patients.

dbSNP	Variant Type	Chr	CADD Score (Scaled)	DANN Score	FATHMM-XF Prediction	SIFT Prediction	PROVEAN Prediction	gnomAD MAF (European

								**Non-Finnish)**
**rs1801552**	synonymous	16	5.661	0.4494	Benign (high conf.)	Tolerated	Neutral	0.6252
**rs9929218**	intron_variant	16	2.120	0.6168	Benign (high conf.)	NA	NA	0.2859
**rs33969373**	synonymous	16	0.549	0.8224	Benign (high conf.)	Tolerated	Neutral	0.007797
**rs2229044**	synonymous	16	7.155	0.7629	Benign (high conf.)	Tolerated	Neutral	0.007156
**rs33964119**	synonymous	16	1.320	0.8216	Benign (high conf.)	Tolerated	Neutral	0.02579
**rs35741240**	synonymous	16	0.029	0.6151	Benign (high conf.)	Tolerated	Neutral	0.004466
**rs761309816**	disruptive_inframe_ deletion	16	NA	NA	NA	NA	NA	NA
**rs61747636**	synonymous	16	8.601	0.785	Benign (high conf.)	Tolerated	Neutral	0.002268
**rs1801023**	synonymous	16	1.198	0.5807	Benign (high conf.)	Tolerated	Neutral	0.004145
**rs1801025**	synonymous	16	7.508	0.9873	Benign	Tolerated	Neutral	0.0007044

NA: not applicable.

© 2020 by the authors. Submitted for possible open access publication under the terms and conditions of the Creative Commons Attribution (CC BY) license (http://creativecommons.org/licenses/by/4.0/).
